# Correction: Gut microbiota signatures of the three Mexican primate species, including hybrid populations

**DOI:** 10.1371/journal.pone.0345160

**Published:** 2026-03-17

**Authors:** Diego Zubillaga-Martín, Brenda Solórzano-García, Alfredo Yanez-Montalvo, Arit de León-Lorenzana, Luisa I. Falcón, Ella Vázquez-Domínguez

In the Data Availability statement, the Bioproject ID number is listed incorrectly. The correct ID number is: Bioproject ID PRJNA1212220.

## References

[1] Zubillaga-Martín D, Solórzano-García B, Yanez-Montalvo A, de León-Lorenzana A, Falcón LI, Vázquez-Domínguez E. Gut microbiota signatures of the three Mexican primate species, including hybrid populations. PLoS One. 2025;20(3):e0317657. doi: 10.1371/journal.pone.0317657 40100798 PMC11918351

