# Supplementary material for: Gut microbiota signatures of the three Mexican primate species, including hybrid populations
Source: PLoS One. 2025 Mar 18;20(3):e0317657. doi: 10.1371/journal.pone.0317657 (PMC11918351; doi:10.1371/journal.pone.0317657)
Supplement: S1 Fig — (PDF) [file pone.0317657.s001.pdf]

a)

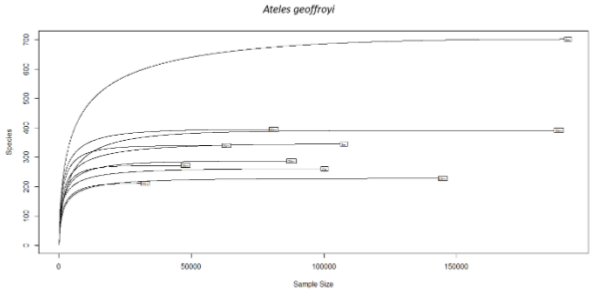

b)

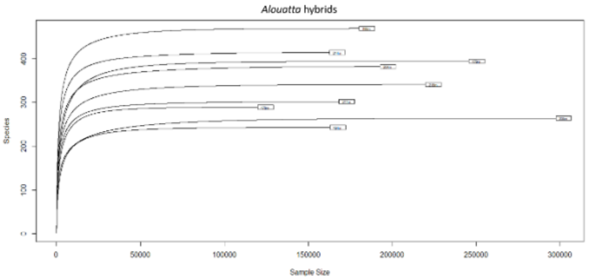

c)

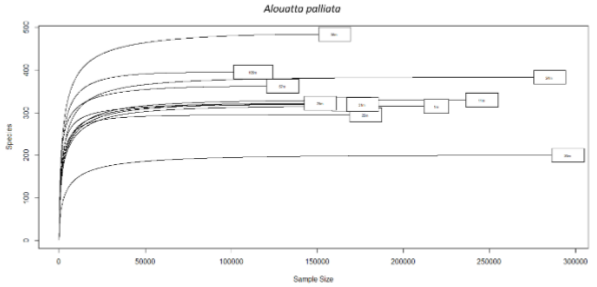

d)

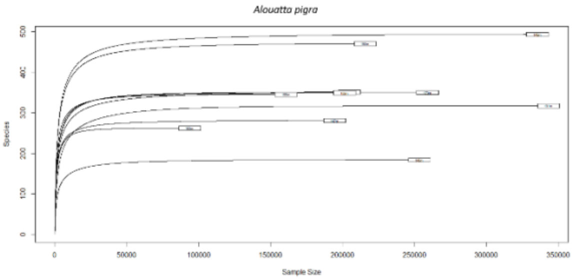

S1 Fig. Accumulation curves for the 16S rDNA V4 sequences of the 40 gut microbiome samples of wild populations of Mexican primates separated by species: **a)** *Ateles geoffroyi*, **b)** *Alouatta* hybrids, **c)** *Alouatta palliata*, **d)** *Alouatta pigra*. All samples reached the asymptote.
