# Supplementary material for: Gut microbiota signatures of the three Mexican primate species, including hybrid populations
Source: PLoS One. 2025 Mar 18;20(3):e0317657. doi: 10.1371/journal.pone.0317657 (PMC11918351; doi:10.1371/journal.pone.0317657)
Supplement: S2 Fig — (PDF) [file pone.0317657.s002.pdf]

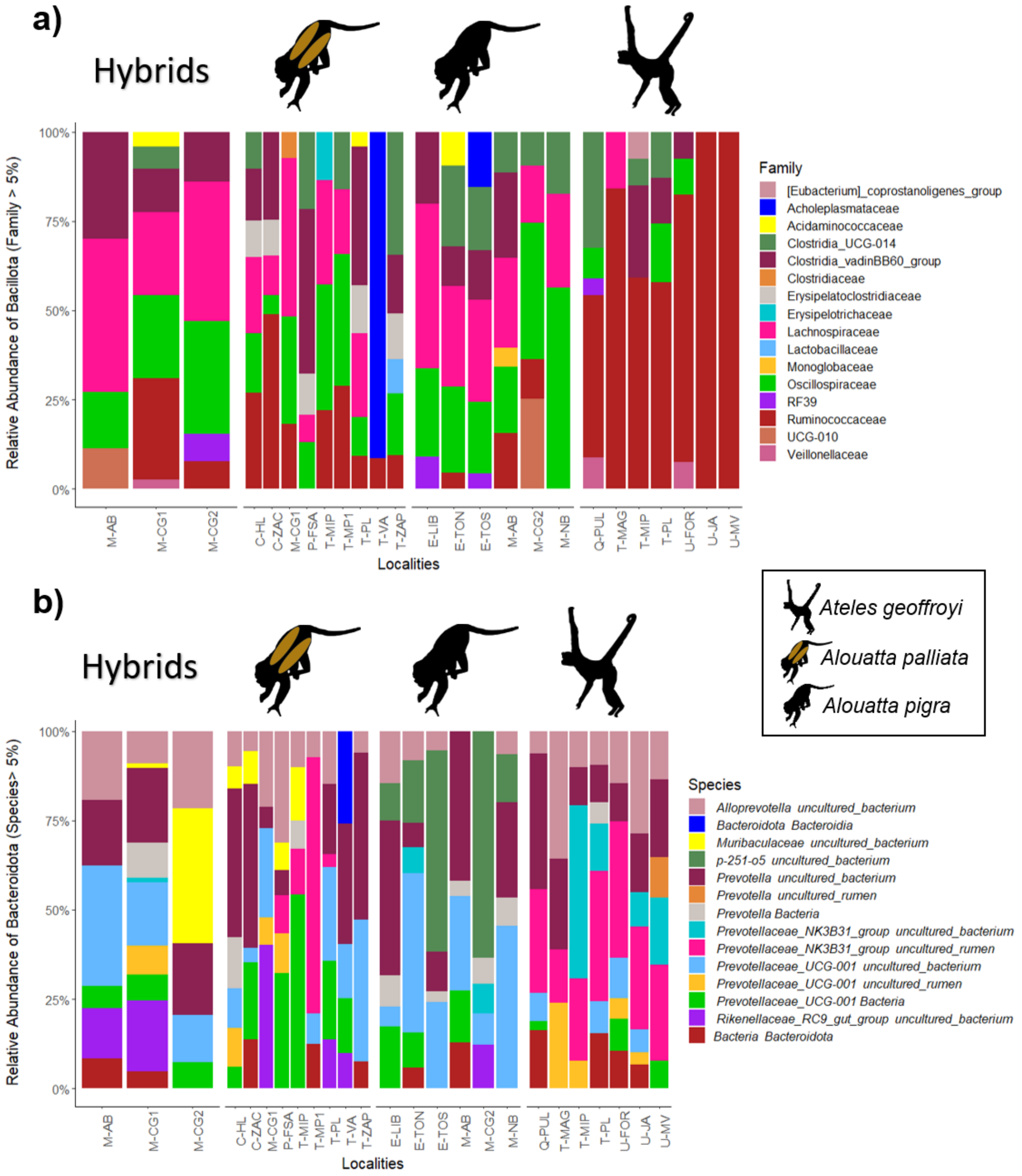

S2 Fig. Relative abundance graphs representing the two most abundant Phyla of gut bacteria diversity from wild populations of Mexican primates, spider monkey (*Ateles geoffroyi*), mantled howler monkey (*Alouatta palliata*), black howler monkey (*A. pigra*), and howlers hybrid individuals. Bacillota represented by family composition filtered by >5% abundance (**a**) and Bacteroidota represented by species composition filtered by >5% abundance (**b**). Each bar represents a sampled locality which are grouped by species. Different families and genera are represented by different colors. Monkey silhouettes obtained from the open source www.phylopic.org, freely available for reuse under Creative Commons licenses.
