# Supplementary material for: Gut microbiota signatures of the three Mexican primate species, including hybrid populations
Source: PLoS One. 2025 Mar 18;20(3):e0317657. doi: 10.1371/journal.pone.0317657 (PMC11918351; doi:10.1371/journal.pone.0317657)
Supplement: S4 Fig — (PDF) [file pone.0317657.s004.pdf]

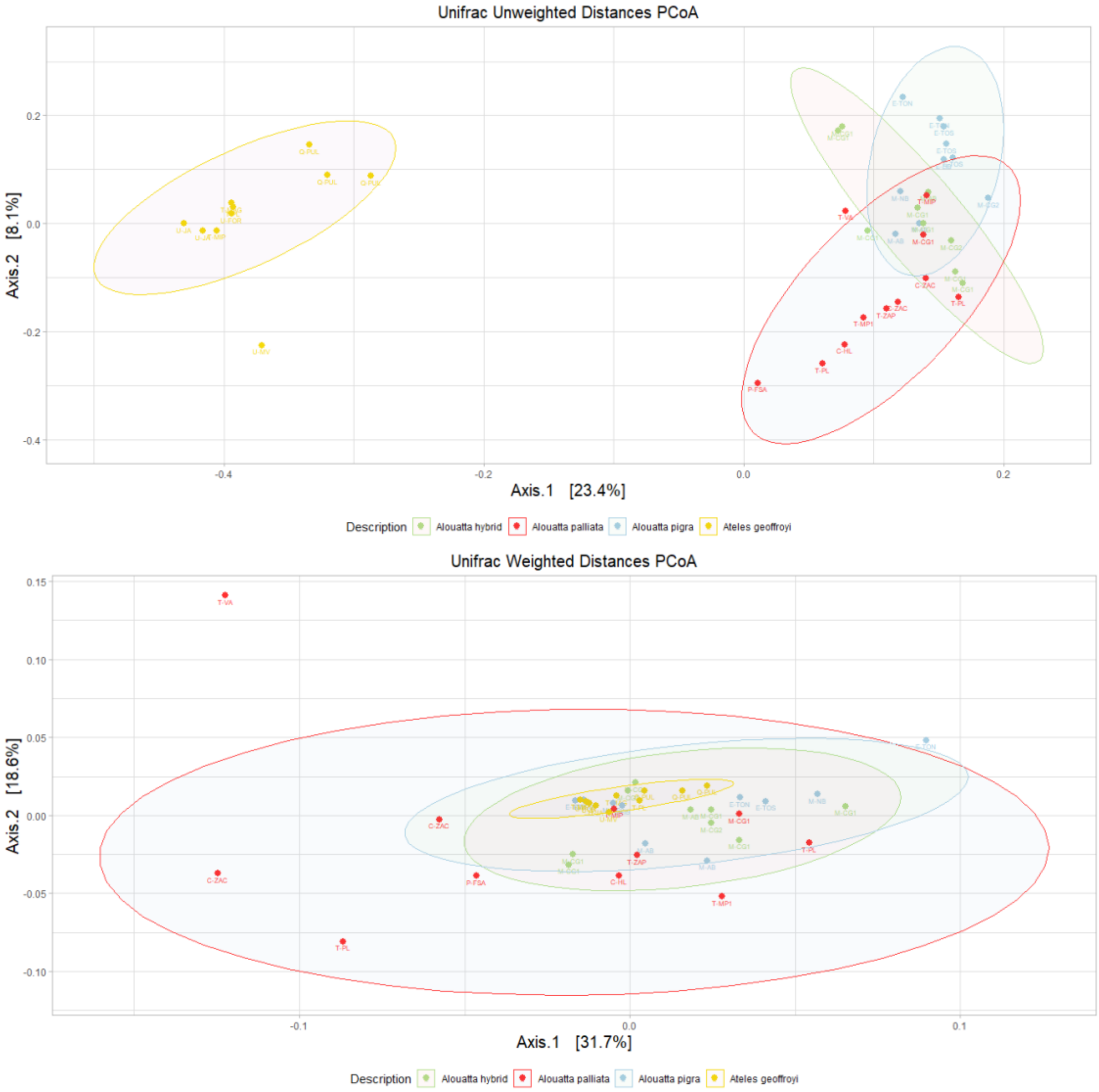

S4 Fig. Principal coordinate analysis (PCoA) based on the rarefied data based on unweighted and weighted Unifrac distance dissimilarities by hosts species of the gut microbiota of three species of Mexican primates: spider monkey (*Ateles geoffroyi*) in yellow, mantled howler monkey (*Alouatta palliata*) in red, black howler monkey (*A. pigra*) in blue, and *Alouatta* hybrid individuals in green.
