# Supplementary material for: Gut microbiota signatures of the three Mexican primate species, including hybrid populations
Source: PLoS One. 2025 Mar 18;20(3):e0317657. doi: 10.1371/journal.pone.0317657 (PMC11918351; doi:10.1371/journal.pone.0317657)
Supplement: S5 Fig — (PDF) [file pone.0317657.s005.pdf]

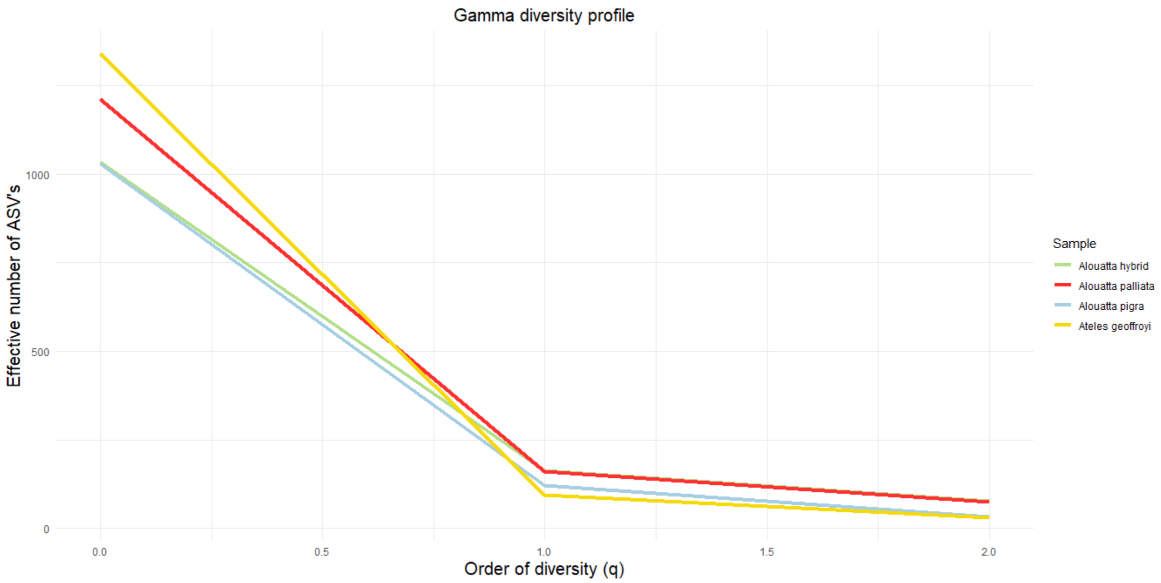

S5 Fig. Gamma diversity based on Hill numbers with q values from 0 to 2 for the three primate species and hybrids; significant statistical differences for q=1 and q=2 between taxa.
