# Supplementary material for: Gut microbiota signatures of the three Mexican primate species, including hybrid populations
Source: PLoS One. 2025 Mar 18;20(3):e0317657. doi: 10.1371/journal.pone.0317657 (PMC11918351; doi:10.1371/journal.pone.0317657)
Supplement: S6 Fig — (PDF) [file pone.0317657.s006.pdf]

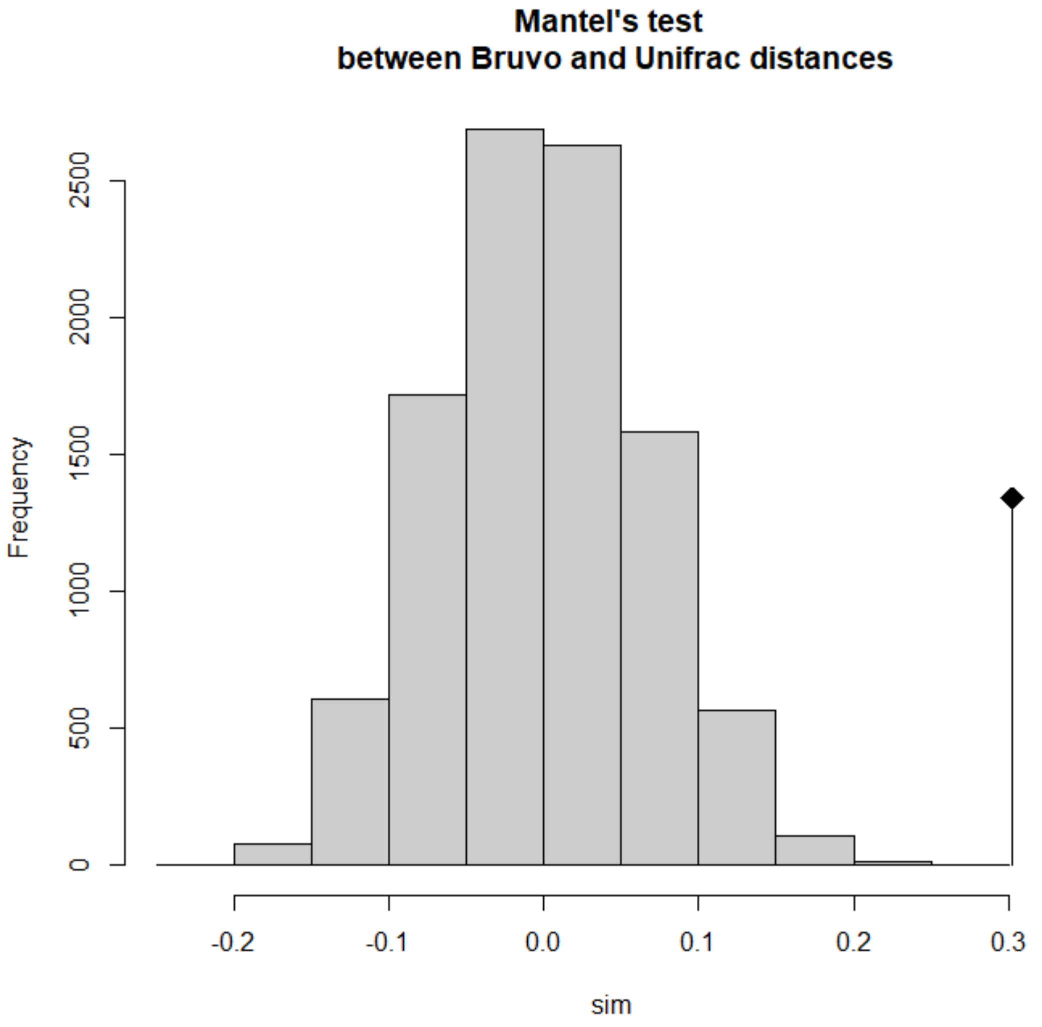

S6 Fig. Mantel test graph results for the correlation between gut microbiota dissimilarities (Unifrac unweighted distance matrix) and genetic dissimilarities (Bruvo's genetic distance matrix) of 30 wild Mexican howler *Alouatta palliata* and *A. pigra* individuals and their hybrids.
