# Supplementary material for: Gut microbiota signatures of the three Mexican primate species, including hybrid populations
Source: PLoS One. 2025 Mar 18;20(3):e0317657. doi: 10.1371/journal.pone.0317657 (PMC11918351; doi:10.1371/journal.pone.0317657)
Supplement: S1 Table — (PDF) [file pone.0317657.s007.pdf]

Table S1. Sampling localities for the wild populations of the three Mexican primate species studied. Geographical region, name of the locality with corresponding code, number of individuals per species (Apa: *Alouatta palliata*; Api: *Alouatta pigra*; Ahy: *Alouatta* hybrids; Ag: *Ateles geoffroyi*), and vegetation type are shown. Vegetation type (Veg) classified as TEF: tall evergreen tropical forest; SDF: semi-deciduous tropical forest; MEF: medium evergreen forest; MSV: mature secondary vegetation.

**Region**	**Locality**	**Code**	**Apa**	**Api**	**Ahy**	**Ag**	**Veg**
Los Tuxtlas	Montepío	MP1	1	-	-	-	TEF
	Magallanes	MAG	-	-	-	1	TEF
	Mirador Pilapa	MIP	1	-	-	1	TEF
	Playa	PL	2	-	-	1	TEF
	Zapoapan	ZAP	1	-	-	-	MEF
	La Valentina	VA	1	-	-	-	TEF
Uxpanapa	El Fortuño	FOR	-	-	-	1	TEF
	El Jaguar	JA	-	-	-	2	TEF
	Murillo Vidal	MV	-	-	-	1	TEF
Comalcalco	Hacienda la Luz	HL	1	-	-	-	MSV
	Zona Arqueológica	ZAC	2	-	-	-	MSV
Pichucalco	Finca Santa Ana	FSA	1	-	-	-	TEF
Macuspana	Nicolas Bravo	NB	-	1	-	-	MSV
	Carlos Green 1	CG1	1	-	7	-	MSV
	Carlos Green 2	CG2	-	1	1	-	MSV
	Agua Blanca	AB	-	2	1	-	TEF
Escárcega	El Tormento Norte	TON	-	2	-	-	SDF
	El Tormento Sur	TOS	-	3	-	-	SDF
	La Libertad	LIB	-	1	-	-	SDF
Punta Laguna	Punta Laguna	PLAG	-	-	-	3	SDF
