# Supplementary material for: Gut microbiota signatures of the three Mexican primate species, including hybrid populations
Source: PLoS One. 2025 Mar 18;20(3):e0317657. doi: 10.1371/journal.pone.0317657 (PMC11918351; doi:10.1371/journal.pone.0317657)
Supplement: S2 Table — (PDF) [file pone.0317657.s008.pdf]

Table S2. Denoising statistics after quality filtering for each of the 40 samples. Spp: species; Api: *Alouatta pigra*; Apa: *Alouatta palliata*; Ag: *Ateles geoffroyi*; Hy: *Alouatta* hybrid individual.

Sample ID	Spp	Input	Filtered	% input passed filter	Denoised	Merged	% of input merged	Non-chimeric	% of input non-chimeric
101m	Api	551109	426184	77.33	422392	406309	73.73	343332	62.3
105m	Api	192695	161036	83.57	158163	147385	76.49	112672	58.47
11m	Apa	396399	329303	83.07	325635	308590	77.85	245716	61.99
147m	Api	387386	323489	83.51	318693	295071	76.17	194505	50.21
148m	Api	449426	337408	75.08	335205	323327	71.94	253511	56.41
149m	Api	570821	495337	86.78	486942	456206	79.92	335688	58.81
14m	Ag	105902	51144	48.29	50257	48016	45.34	32620	30.8
150m	Api	363065	283868	78.19	280392	264926	72.97	204770	56.4
152m	Api	352811	287810	81.58	283135	264356	74.93	201443	57.1
155m	Api	272089	210919	77.52	207574	194700	71.56	160742	59.08
15m	Ag	404767	320799	79.26	318498	304875	75.32	188768	46.64
164m	Hy	306521	250158	81.61	247326	233348	76.13	167536	54.66
173m	Api	462665	376235	81.32	371240	352939	76.28	259152	56.01
176m	Apa	287475	237131	82.49	233920	223348	77.69	169843	59.08
178m	Hy	224670	186164	82.86	183163	173603	77.27	124537	55.43
179m	Hy	456756	381377	83.5	376112	348304	76.26	250373	54.82
180m	Api	398035	341751	85.86	334823	308701	77.56	215856	54.23
183m	Api	164774	137917	83.7	135926	129179	78.4	93707	56.87
198m	Hy	342007	289220	84.57	283398	259788	75.96	184828	54.04
1m	Apa	340426	296569	87.12	293398	279297	82.04	219155	64.38
204m	Hy	336271	283552	84.32	278913	263938	78.49	197393	58.7
205m	Hy	512411	424727	82.89	420184	398948	77.86	302088	58.95
20m	Apa	434553	383969	88.36	381017	369619	85.06	295629	68.03
211m	Hy	309365	248479	80.32	245043	231604	74.86	172957	55.91
214m	Hy	285388	229716	80.49	225664	211961	74.27	167349	58.64
215m	Hy	413193	318727	77.14	313602	292989	70.91	224532	54.34
24m	Apa	492738	385284	78.19	380202	361094	73.28	284947	57.83
25m	Apa	309267	248181	80.25	244306	230379	74.49	178051	57.57
31m	Apa	290451	241392	83.11	238834	227429	78.3	176206	60.67
33m	Ag	180991	127677	70.54	126311	119149	65.83	87772	48.5
36m	Apa	276780	215425	77.83	211294	197303	71.29	159997	57.81
38m	Ag	103248	75043	72.68	74126	69742	67.55	47712	46.21
39m	Ag	150782	103597	68.71	101364	94728	62.82	63130	41.87
3m	Ag	234813	160826	68.49	157809	147306	62.73	107576	45.81
40m	Ag	153558	122557	79.81	119612	109409	71.25	81009	52.75
41m	Ag	273857	225107	82.2	223044	214371	78.28	144974	52.94
57m	Apa	226733	184701	81.46	180277	167317	73.79	129856	57.27
5m	Ag	398534	252226	63.29	247631	235623	59.12	192230	48.23
75m	Apa	282976	231308	81.74	226352	210423	74.36	151742	53.62
7m	Ag	227886	153525	67.37	151351	141843	62.24	100219	43.98
