# Supplementary material for: Gut microbiota signatures of the three Mexican primate species, including hybrid populations
Source: PLoS One. 2025 Mar 18;20(3):e0317657. doi: 10.1371/journal.pone.0317657 (PMC11918351; doi:10.1371/journal.pone.0317657)
Supplement: S3 Table — (PDF) [file pone.0317657.s009.pdf]

Table S3. Amplicon sequence variants (ASVs) identified per host species based on the 'unfiltered dataset' (V4 16S RNA) from 40 individuals of Mexican primate species: 11 *Alouatta palliata*, 10 *A. pigra*, 9 *Alouatta* hybrid individuals and 10 *Ateles geoffroyi*.

**Host**	**Total elements**	**ASVs**
Alouatta palliata<br>*Alouatta pigra*<br>*Alouatta* hybrids<br>*Ateles geoffroyi*	106	UCG-008 uncultured_bacterium, Syntrophococcus Bacteria, Clostridia_UCG-014 Bacteria, p-251-o5 uncultured_bacterium, NK4A214_group Bacteria, Cerasicoccus Bacteria, Bacteria Verrucomicrobiota, Desulfovibrio uncultured_bacterium, Clostridia_UCG-014 uncultured_bacterium, Oscillibacter Bacteria, Rikenellaceae_RC9_gut_group uncultured_bacterium, Olsenella Bacteria, Lachnospiraceae_NK4A136_group uncultured_bacterium, Coprococcus uncultured_bacterium, Subdoligranulum uncultured_bacterium, Oribacterium uncultured_bacterium, [Eubacterium]_ventriosum_group uncultured_bacterium, Colidextribacter uncultured_bacterium, Lachnospiraceae_NK4A136_group Bacteria, Ruminococcus uncultured_bacterium, GCA-900066575 uncultured_bacterium, uncultured uncultured_Acidobacteria, UCG-004 uncultured_bacterium, Ruminococcus Bacteria, Prevotella uncultured_bacterium, Archaea Thermoplasmatota, Faecalibacterium uncultured_bacterium, Muribaculaceae uncultured_bacterium, Clostridia_vadinBB60_group uncultured_bacterium, Clostridia_vadinBB60_group Bacteria, [Eubacterium]_coprostanoligenes_group uncultured_bacterium, Erysipelotrichaceae_UCG-006 uncultured_bacterium, Mitochondria Bacteria, Slackia Bacteria, Gastranaerophilales uncultured_bacterium, [Eubacterium]_siraeum_group uncultured_bacterium, Anaerostipes uncultured_bacterium, Blautia Bacteria, Shuttleworthia uncultured_bacterium, Butyrivibrio uncultured_rumen, NK4A214_group uncultured_bacterium, [Eubacterium]_ruminantium_group Bacteria, Lachnospiraceae_UCG-001 uncultured_bacterium, Bacteria Bacteroidota, Colidextribacter uncultured_rumen, uncultured uncultured_rumen, Family_XIII_AD3011_group uncultured_bacterium, Anaeroplasma uncultured_bacterium, Tyzzerella Bacteria, RF39 Bacteria, uncultured Bacteria, Lachnospiraceae_NK3A20_group uncultured_bacterium, Lachnospiraceae_UCG-010 uncultured_bacterium, Raoultibacter Bacteria, Blautia uncultured_bacterium, Monoglobus uncultured_bacterium, Roseburia Bacteria, Roseburia uncultured_bacterium, Prevotellaceae_NK3B31_group uncultured_bacterium, UCG-005 uncultured_bacterium, Methanosphaera uncultured_archaeon, Candidatus_Methanomethylophilus Archaea, Lachnospiraceae_UCG-008 uncultured_bacterium, uncultured uncultured_bacterium, Bacteria Firmicutes, Tyzzerella uncultured_bacterium, Family_XIII_UCG-001 uncultured_bacterium, Clostridia_UCG-014 uncultured_rumen, Chloroplast Bacteria, Lachnospiraceae_FCS020_group Bacteria, Desulfovibrio Bacteria, Tyzzerella uncultured_rumen, Mogibacterium Bacteria, Solobacterium uncultured_bacterium, Collinsella uncultured_bacterium, Actinobacteriota Coriobacteriia, Agathobacter uncultured_bacterium, Butyricicoccus uncultured_bacterium, Slackia uncultured_bacterium, Campylobacter Bacteria, Alloprevotella uncultured_bacterium, CAG-352 uncultured_bacterium, Tannerella Bacteria, Lachnospiraceae_ND3007_group uncultured_bacterium, Prevotellaceae_UCG-001 Bacteria, Prevotellaceae_UCG-001 uncultured_bacterium, Prevotella Bacteria, [Eubacterium]_xylanophilum_group uncultured_bacterium, Clostridia Bacteria, Sutterella uncultured_bacterium, Lachnoclostridium Bacteria, Bacteria Actinobacteriota, Helicobacter Helicobacter_sp., Gastranaerophilales Bacteria, Lachnospiraceae_UCG-008 Bacteria, RB41 uncultured_bacterium, [Eubacterium]_ruminantium_group

		uncultured_bacterium, Fretibacterium uncultured_rumen, Christensenellaceae_R-7_group uncultured_bacterium, Bacteroides uncultured_bacterium, Sarcina uncultured_bacterium, Bacteroidota Bacteroidia, Marvinbryantia Bacteria, Prevotellaceae_NK3B31_group uncultured_rumen, UCG-010 uncultured_bacterium, RF39 uncultured_bacterium
Alouatta palliata<br>*Alouatta pigra*<br>*Alouatta* hybrids	24	Syntrophomonas uncultured_bacterium, Oxalobacter Bacteria, Escherichia-Shigella Escherichia_coli, [Eubacterium]_hallii_group uncultured_rumen, Oscillibacter uncultured_bacterium, Family_XIII_AD3011_group Bacteria, Erysipelotrichaceae_UCG-002 Erysipelotrichaceae_bacterium, Candidatus_Methanomethylophilus uncultured_archaeon, Lachnoclostridium uncultured_bacterium, Incertae_Sedis uncultured_bacterium, Akkermansia uncultured_bacterium, Marvinbryantia uncultured_bacterium, Firmicutes Clostridia, Caproiciproducens Bacteria, Phascolarctobacterium Bacteria, Coriobacteriaceae_UCG-002 uncultured_bacterium, Candidatus_Saccharimonas uncultured_rumen, Streptococcus uncultured_bacterium, Hungateiclostridium Hungateiclostridium_thermocellum, UCG-002 Bacteria, [Eubacterium]_nodatum_group Bacteria, Howardella uncultured_rumen, [Eubacterium]_nodatum_group uncultured_rumen, Ruminiclostridium uncultured_bacterium
Alouatta palliata<br>*Alouatta pigra*<br>*Ateles geoffroyi*	7	Reyranella Bacteria, Candidatus_Udaeobacter Bacteria, Candidatus_Udaeobacter uncultured_bacterium, Lachnospiraceae_UCG-001 Bacteria, Bacteria Proteobacteria, MND1 uncultured_bacterium, Candidatus_Xiphinematobacter uncultured_bacterium
Alouatta palliata<br>*Ateles geoffroyi*<br>*Alouatta* hybrids	12	Allorhizobium-Neorhizobium-Pararhizobium-Rhizobium Bacteria, Elusimicrobium uncultured_bacterium, Prevotellaceae_UCG-001 uncultured_rumen, Anaeromyxobacter uncultured_bacterium, Latescibacterota uncultured_bacterium, WD2101_soil_group uncultured_bacterium, Rokubacteriales uncultured_bacterium, Alistipes uncultured_bacterium, Mucispirillum uncultured_bacterium, Erysipelotrichaceae_UCG-009 uncultured_bacterium, Atopobium Bacteria, Enterobacter Bacteria
Alouatta pigra<br>*Ateles geoffroyi*<br>*Alouatta* hybrids	5	Bacteria Bacteria, Lachnospiraceae_XPB1014_group Bacteria, Lachnospira uncultured_bacterium, Alphaproteobacteria Bacteria, [Bacteroides]_pectinophilus_group uncultured_bacterium
Alouatta palliata<br>*Alouatta pigra*	9	Christensenellaceae_R-7_group Bacteria, Methanobrevibacter uncultured_archaeon, A4b uncultured_bacterium, Akkermansia Bacteria, F0058 uncultured_bacterium, RB41 Bacteria, Lachnospiraceae_ND3007_group Bacteria, KD3-93 Bacteria, Shuttleworthia Bacteria
Alouatta palliata<br>Alouatta hybrids	8	Intestinimonas Bacteria, Barnesiella uncultured_bacterium, Lachnospiraceae_FCS020_group uncultured_bacterium, Defluviitaleaceae_UCG-011 uncultured_bacterium, UCG-002 uncultured_bacterium, vadinBE97 Bacteria, Parasutterella uncultured_bacterium, Vicinamibacteraceae uncultured_bacterium
Alouatta palliata<br>*Ateles geoffroyi*	36	Staphylococcus Bacteria, Pseudonocardia uncultured_bacterium, Burkholderia-Caballeronia-Paraburkholderia Burkholderia_multivorans, Pseudomonas Bacteria, Lactococcus Bacteria, KD4-96 uncultured_bacterium, NB1-j uncultured_bacterium, Lactobacillus Lactobacillus_plantarum, Bacillus uncultured_bacterium, Bradyrhizobium Bradyrhizobium_sp., Pir4_lineage uncultured_bacterium, Prevotella uncultured_rumen, Achromobacter Bacteria, Tetratrichomonas Eukaryota, Flavobacterium uncultured_bacterium, Kurthia uncultured_bacterium, SH-PL14

		Bacteria, 67-14 uncultured_bacterium, Gammaproteobacteria Bacteria, Pla4_lineage Bacteria, Polycyclovorans uncultured_bacterium, Enterococcus Bacteria, Acidibacter uncultured_bacterium, 0319-6G20 Bacteria, Subgroup_2 uncultured_bacterium, Burkholderia-Caballeronia-Paraburkholderia Bacteria, 11-24 uncultured_bacterium, Pedosphaeraceae uncultured_bacterium, Alloprevotella Bacteria, Vicinamibacteraceae Bacteria, OM190 uncultured_bacterium, SBR1031 uncultured_bacterium, Fimbriimonadaceae uncultured_bacterium, Delftia Bacteria, BIrii41 Bacteria, Acinetobacter Bacteria
Alouatta pigra<br>*Alouatta* hybrids	6	UBA1819 Bacteria, Prevotella uncultured_organism, Incertae_Sedis Bacteria, UCG-005 uncultured_rumen, Monoglobus Bacteria, Acetitomaculum Bacteria
Alouatta pigra<br>*Ateles geoffroyi*	9	SM2D12 Bacteria, Chryseolinea Bacteria, Mycoplasma Bacteria, Nitrososphaeraceae Archaea, Bryobacter Bacteria, Selenomonas Bacteria, Gastranaerophilales uncultured_rumen, Unassigned Unassigned, Pedobacter Bacteria
Alouatta hybrids<br>*Ateles geoffroyi*	8	Enterorhabdus uncultured_bacterium, Rhodoplanes Bacteria, Sphingomonas uncultured_bacterium, Devosia uncultured_bacterium, RF39 uncultured_rumen, Haliangium uncultured_bacterium, Coprobacter Bacteria, Brevundimonas Bacteria
Alouatta palliata	59	WCHB1-41 Bacteria, AKYH767 uncultured_bacterium, [Eubacterium]_coprostanoligenes_group Bacteria, Mitochondria Solanum_melongena, PLTA13 uncultured_bacterium, FD2005 uncultured_Lachnospiraceae, Subgroup_22 uncultured_bacterium, Abditibacterium uncultured_bacterium, Ignavibacterium Bacteria, Shimazuella Bacteria, IS-44 uncultured_bacterium, Treponema Bacteria, KF-JG30-B3 uncultured_bacterium, Rhizobacter uncultured_bacterium, vadinHA49 uncultured_bacterium, Anaerofilum uncultured_bacterium, Vicinamibacteraceae uncultured_Acidobacteria, Candidatus_Omnitrophus uncultured_bacterium, Dongia uncultured_bacterium, bacteriap25 Bacteria, Streptomyces Streptomyces_sp., Gitt-GS-136 uncultured_bacterium, Zixibacteria uncultured_bacterium, Subgroup_25 uncultured_bacterium, CCD24 uncultured_bacterium, Pla4_lineage uncultured_bacterium, Sporichthya Bacteria, Nakamurella uncultured_bacterium, KF-JG30-C25 uncultured_bacterium, TRA3-20 uncultured_bacterium, SM1A02 uncultured_bacterium, OM190 Bacteria, Synergistes uncultured_organism, Catellatospora Bacteria, Iamia Bacteria, Weissella Weissella_paramesenteroides, Pediococcus Pediococcus_pentosaceus, EPR3968-O8a-Bc78 Bacteria, ABY1 Bacteria, RB41 uncultured_Acidobacteria, Nordella uncultured_bacterium, Campylobacter Campylobacter_sputorum, R7C24 metagenome, CCM11a Bacteria, Cloacibacillus Bacteria, Rhodococcus Rhodococcus_erythropolis, BSV26 uncultured_bacterium, Serratia Bacteria, Mobiluncus Mobiluncus_mulieris, Ellin516 Bacteria, Escherichia-Shigella Bacteria, Lineage_IIa Bacteria, Chryseobacterium uncultured_bacterium, Haemophilus uncultured_bacterium, Mucilaginibacter Bacteria, Pedomicrobium uncultured_bacterium, Sulfurifustis Bacteria, Candidatus_Koribacter uncultured_bacterium, Pseudomonas Pseudomonas_putida
Alouatta pigra	20	Mitochondria Botryosphaeria_dothidea, Chloroplast uncultured_bacterium, Pseudoflavitalea Bacteria, Cytophaga Bacteria, Fusicatenibacter uncultured_bacterium, Mesorhizobium Bacteria, Nocardia Bacteria, Verrucomicrobiae Bacteria, Conexibacter Bacteria, Bosea uncultured_bacterium, NK4A214_group uncultured_rumen, Gaiella uncultured_bacterium, Victivallis Bacteria, AKYG587 uncultured_bacterium, Coriobacteriia Bacteria, Lachnospiraceae_NK3A20_group Bacteria, Firmicutes Bacteria, Incertae_Sedis uncultured_organism, Gordonibacter

		uncultured_Gordonibacter, UCG-010 Bacteria
Alouatta hybrids	18	Lactobacillus uncultured_bacterium, mle1-27 Bacteria, Colidextribacter Bacteria, UCG-005 Bacteria, Pelosinus uncultured_bacterium, Erysipelotrichaceae_UCG-002 uncultured_bacterium, Luteimonas Bacteria, Eisenbergiella Bacteria, Bilophila uncultured_bacterium, Subgroup_17 uncultured_bacterium, Phenylobacterium uncultured_bacterium, Lachnospiraceae_XPB1014_group uncultured_bacterium, Bacteroides Bacteroides_ovatus, Armatimonadales uncultured_bacterium, Mogibacterium uncultured_bacterium, Alistipes Bacteria, Novosphingobium uncultured_bacterium, Pseudoxanthomonas Bacteria
Ateles geoffroyi	173	Butyrivibrio uncultured_bacterium, Porphyrobacter Bacteria, Luteimonas uncultured_bacterium, UCG-004 Bacteria, Nocardioides Bacteria, Pirellula Bacteria, Altererythrobacter Bacteria, Latescibacterota Bacteria, MB-A2-108 uncultured_bacterium, Fimbriimonadaceae Bacteria, BIrii41 uncultured_bacterium, Fluviicola Bacteria, Acholeplasma uncultured_bacterium, Solibacillus uncultured_bacterium, Acinetobacter Acinetobacter_sp., Herpetosiphon Bacteria, Megasphaera uncultured_bacterium, Fibrobacterota Fibrobacteria, Stenotrophomonas uncultured_bacterium, Lautropia Bacteria, Actinotalea Bacteria, Bacteroidetes_BD2-2 uncultured_bacterium, Vagococcus Bacteria, F082 Bacteria, Subgroup_10 uncultured_bacterium, Peredibacter Bacteria, Prevotellaceae_NK3B31_group Bacteria, Blyi10 Bacteria, Kapabacteriales uncultured_bacterium, Rhodobacter uncultured_bacterium, Flavobacterium Flavobacterium_sp., Pseudolabrys Bacteria, Taibaiella uncultured_bacterium, Pelagibacterium Bacteria, Ochrobactrum Bacteria, Bacteria Chloroflexi, Kineosporia Bacteria, Dysgonomonas Bacteria, uncultured uncultured_organism, Succinivibrio Bacteria, Sphingobium Bacteria, Parcubacteria uncultured_bacterium, Cohnella Bacteria, NS9_marine_group Bacteria, Butyrivibrio Bacteria, Massilia Bacteria, Bifidobacterium uncultured_bacterium, Blastocatella Bacteria, Kosakonia Bacteria, Anaeromyxobacter Bacteria, Adlercreutzia Bacteria, Candidatus_Riegeria Bacteria, Methanobrevibacter Archaea, Adhaeribacter Bacteria, Erysipelotrichaceae_UCG-009 Bacteria, Chthonomonadales uncultured_bacterium, Rikenellaceae_RC9_gut_group uncultured_rumen, Aurantisolimonas uncultured_bacterium, Acidimicrobiia Bacteria, Ramlibacter Bacteria, Paracoccus Bacteria, Sutterella Bacteria, Timonella uncultured_bacterium, Bacteroidetes_VC2.1_Bac22 Bacteria, Segetibacter uncultured_bacterium, Mitochondria Triticum_aestivum, Leucobacter uncultured_bacterium, Sphingomonas Bacteria, Sphingobacterium Bacteria, Holdemanella uncultured_bacterium, Terrimonas uncultured_bacterium, Faecalibacterium Bacteria, Pseudobutyrivibrio Bacteria, Streptomyces Bacteria, [Eubacterium]_eligens_group Bacteria, Lineage_IIb uncultured_bacterium, Olsenella uncultured_bacterium, Novosphingobium Bacteria, Phaselicystis uncultured_bacterium, Cellvibrio uncultured_bacterium, Sphingobacterium uncultured_bacterium, Succinivibrio uncultured_bacterium, Pseudomonas Pseudomonas_aeruginosa, Algoriphagus Bacteria, Hirschia Bacteria, Mycobacterium Bacteria, Termite_Treponema_cluster Bacteria, Corynebacterium uncultured_bacterium, Corynebacterium Bacteria, BIrii41 metagenome, IMCC26256 uncultured_bacterium, Actinoplanes Bacteria, Lactococcus Lactococcus_lactis, Ferruginibacter uncultured_bacterium, Cellvibrio Bacteria, Nocardioides uncultured_bacterium, Asteroleplasma uncultured_bacterium, Erysipelothrix uncultured_bacterium, Xanthomonas Bacteria, Leuconostoc Leuconostoc_pseudomesenteroides, Fibrobacterota Bacteria, Chthoniobacter Bacteria, Candidatus_Solibacter uncultured_bacterium, Terrimicrobium uncultured_bacterium,

		Pseudonocardia Pseudonocardia_sp., GOUTA6 Bacteria, Roseomonas Bacteria, A4b Bacteria, Comamonas Bacteria, Pedomicrobium Bacteria, Stenotrophomonas Stenotrophomonas_maltophilia, Subgroup_7 uncultured_bacterium, Lachnoclostridium uncultured_organism, Solirubrobacter Bacteria, Bacteria Cyanobacteria, Bdellovibrio uncultured_bacterium, Weeksella Bacteria, Gemella uncultured_bacterium, Cyanobacteriia Bacteria, Actinobacteriota Actinobacteria, Paenibacillus uncultured_bacterium, Cyanobacteria Cyanobacteriia, Weissella Bacteria, Blfdi19 uncultured_bacterium, Bosea Bacteria, Bacillus Bacillus_sp., Glutamicibacter Arthrobacter_sp., AD3 uncultured_bacterium, Lampropedia Lampropedia_hyalina, Phycicoccus Bacteria, env.OPS_17 uncultured_bacterium, Arenimonas uncultured_bacterium, Leucobacter Bacteria, Aerococcus Bacteria, Glutamicibacter uncultured_bacterium, Elizabethkingia Bacteria, Paracoccus uncultured_bacterium, Allorhizobium-Neorhizobium-Pararhizobium-Rhizobium Rhizobium_sp., Microvirga Bacteria, Catenibacterium uncultured_bacterium, Chloroflexi Bacteria, OM27_clade uncultured_bacterium, Hypotrichomonas Eukaryota, Streptococcus Bacteria, Lachnoclostridium Lachnoclostridium_phytofermentans, OLB14 uncultured_bacterium, Lachnospiraceae Bacteria, Paenibacillus Paenibacillus_sp., Dyadobacter Bacteria, Ketogulonicigenium Ketogulonicigenium_vulgare, Acholeplasma Bacteria, Comamonas uncultured_bacterium, Leadbetterella uncultured_bacterium, SM1A02 metagenome, Subgroup_5 uncultured_bacterium, Bacteria Acidobacteriota, [Eubacterium]_eligens_group uncultured_bacterium, Selenomonas uncultured_rumen, DNF00809 Bacteria, Asticcacaulis Bacteria, Chryseobacterium Bacteria, Bacillus Bacteria, Serratia Serratia_sp., Stenotrophomonas Bacteria, Fluviicola uncultured_bacterium, Paenibacillus Bacteria, Nitrososphaeraceae uncultured_archaeon, Pedosphaeraceae Bacteria, 11-24 Bacteria, AKYH767 Bacteria, RBG-13-54-9 uncultured_bacterium, Flavobacterium Bacteria, Caulobacter Bacteria
