# Supplementary material for: Gut microbiota signatures of the three Mexican primate species, including hybrid populations
Source: PLoS One. 2025 Mar 18;20(3):e0317657. doi: 10.1371/journal.pone.0317657 (PMC11918351; doi:10.1371/journal.pone.0317657)
Supplement: Attachment — Submitted filename: Response to Reviewers.pdf [file pone.0317657.s011.pdf]

Response to reviewers
We thank the thoughtful and constructive feedback of the reviewers, which helped us to improve the manuscript throughout.

Academic Editor
Comments to the Author:
Both reviewers agreed that this study provides valuable information for the research community. However, both noted a number of issues regarding experimental setup, data analysis, interpretation, and discussion that need to be adequately addressed before further consideration can be made..
We thank the Academic Editor and include here a 'Point-by-point response', where we carefully answer every comment and suggestion made. To facilitate locating the changes we made, they are indicated with track changes in the file named “Revised Manuscript with Track Changes”, and with blue lettering in the responses below in this document.

Reviewers' comments to the Author

The PLOS Data policy requires authors to make all data underlying the findings described in their manuscript fully available without restriction, with rare exception (please refer to the Data Availability Statement in the manuscript PDF file). The data should be provided as part of the manuscript or its supporting information, or deposited to a public repository. For example, in addition to summary statistics, the data points behind means, medians and variance measures should be available. If there are restrictions on publicly sharing data—e.g. participant privacy or use of data from a third party—those must be specified.
Reviewer #1: No
Reviewer #2: No
Response: We uploaded the sequence data and now include the following in the Data Availability Statement: “The 16S raw sequence data are publicly available in NCBI under the Bioproject number ID PRJNA1189536. Metadata and methods for analyses are available in the main text and in the Supporting Information”

Reviewer #1:
Summary: The study presented in “Gut microbiota signatures of the three Mexican primate species, including hybrid populations” by Zubillaga and colleagues examines the gut microbiome composition of wild populations of the three Mexican primates and hybrid populations. Their results revealed distinct microbiome compositions in the 3 NHP species strongly shaped by host specific factors.

While I believe this is an important work and is a good fit for the PLOS ONE journal, there are number of minor concerns I would like the authors to address.

Minor Concerns:
Sample size: A total sample size of 40 with 9-11 samples might reduce the power the statistical analysis. Did the authors do a power analysis to determine if these samples sizes are sufficient?
Response: We appreciate the reviewer’s comment.
Yes we did. We first calculated accumulation curves by host species, confirming that the 40 samples reached an asymptote. We estimated Permutational multivariate analysis of variance (Permanova), a method to test for significant differences in microbial community composition between different groups,

which rendered significant differences between all pairs of species (results shown in Table 1). A global anosim test (Analysis of similarities) was also significant (R = 0.7354; p = 1e-04) (not included in the ms).

Rarefaction: This is a crucial step in microbiome studies which addresses biases that arise from unequal sampling depths allowing for fair comparisons between samples. Given that all 40 samples had more than 20,000 reads, why did the authors choose not to perform rarefaction. With such high number of reads per sample, they would not have to drop any samples even with rarefaction. Could the authors check how rarefaction would impact the results?
Response: Indeed, based on the high number of reads per sample we obtained and that no sample would need to be eliminated, we decided not to perform a rarefaction process.
However, based on the reviewer's concern, for this revised version we did (lines 212-214): "We also did rarefaction without sample replacement using a rarefaction depth of 90% of the minimum sample depth (28,883.7 reads per sample) ('rarefied dataset')."

And, for comparative purposes, we also performed the same PCoA analysis using the rarefied dataset, based on unweighted and weighted Unifrac distance dissimilarities to assess prokaryote community structure among the primate species and hybrids and to visualize abundance effects. Results showed that patterns did not differ for the species or the hybrids (see Supplementary Fig 4).

Reviewer #2:
In this brief report the authors provide results from a study of gut microbiota from three wild primate populations of black and mantled howler monkeys (Alouatta palliata and Alouatta pigra, respectively) and spider monkeys (Ateles geoffroyi) from different regions in Mexico. They also examined hybrid offspring between the groups. The methodology used was genomic DNA extraction, followed by 16S rRNA gene amplification and sequencing of the V4 region using Illumina MiSeq platform. The resulting reads were processed into ASVs with QIIME2, followed by SILVA classification and alignment with MAFFT and FastTree, phyloseq, rarefaction, and PCoA analysis. The authors examined the interspecific and intraspecific gut microbial diversity, abundance, and composition profiles of 40 individuals from the three populations. They found that interspecific differences of gut microbial diversity were higher than intraspecific differences.
They found that hybrids had microbial diversities in between that of their parental species, but also showed some unique microbial taxa. They also found evidence that microbial compositions were affected by environment, geographic distribution, and host genetics. Overall, the results are interesting and so long as the authors make the sequences available to the community through public sequence databases the data will contribute to our understanding of the microbial communities of primates. However, there some issues that if adequately addressed would significantly improve this manuscript:

1. There does not appear to have been any power analysis performed to assess the statistical significance of the results. Is a total sample size of 40 individuals with 3 species groups and several hybrid groups provide sufficient power? This is particularly a concern considering that the authors mention their results differ from other reported studies and that it might be due to sample size differences or due to temporal differences in sampling. Not knowing the more likely contributor to the differences lowers the overall impact of the study.
Response: We thank the reviewer's comment.
Yes we did. We first calculated accumulation curves by host species, confirming that the 40 samples reached an asymptote. We estimated Permutational multivariate analysis of variance (Permanova), a method to test for significant differences in microbial community composition between different groups,

which rendered significant differences between all pairs of species (results shown in Table 1). A global anosim test (Analysis of similarities) was also significant (R = 0.7354; p = 1e-04) (not included in the ms).

2. Line 253 – since the cited study is in preparation (not published), this data should be either included here in this manuscript or excluded completely. There is limited way for a reviewer to make an adequate assessment of the quality of the results described without that data.
Response: We appreciate the reviewer's comment. We respectfully differ in the need of the genetic (microsatellite) data to sustain the validity of our results. We explain why:
- Describing the complete process performed (microsatellite molecular laboratory, amplification, genetic analyses) will imply to present a whole new article (the one that is in fact in preparation to submit as soon as possible but that has taken longer than the microbiome study).
- The mention of the unpublished work is as support of the identity of the nine hybrid individuals we use in the present study; we indicate: "Hybrid individuals were confirmed by population genetics and introgression analyses based on seven microsatellite loci".
- We do not consider that the quality of our results is compromised by not having the microsatellite genetic data available. The results we present in the manuscript, namely the gut microbiota data of the three primate species and the hybrid individuals, were analyzed with a comprehensive set of methodological and analytical tools appropriate for microbiome bioinformatics.
- We could provide a table of the 40 samples with their microsatellite genotypes, but we do not think it will make a difference to the context and robustness of our study and the manuscript presented. We leave for the Academic Editor to decide if we should include it

3. Figure 3 is not very useful. A more comprehensive analysis of the connection of the taxa in each of the samples with the nature of the environment, location, food sources at time of sample collection, dietary preferences, and metabolic implications regarding the taxa present would have been more meaningful. For example, what taxa are shared between the hybrids and their parent groups? Does this make sense with the environmental factors and their diets or locations? More focus on the differences observed in taxa based on some of the demographics would be more informative.
Response: The reviewer is right in pointing out that it would have been meaningful to evaluate the gut microbiota patterns and their association in more detail with aspects of the hosts' environment, location, food sources at time of sample collection, dietary preferences, demographics, and metabolic implications. Nonetheless, we do not have the required data to perform such association assessments in a sound and robust fashion.
We added the following text in our final paragraph, to complement the proposals we indicated for future research (lines 516-519): "Studies should target to obtain detailed information related to the host at the time of sampling (e.g. environmental variables, vegetation type, forest cover, food availability), which will enable us to continue deciphering ecological and evolutionary factors affecting the gut microbiota"

We also disagree with the reviewer on key points. These disagreements might represent misunderstandings on the intent of our paper, thus we provide further clarification and justification below.

We carefully considered what we would be able to assess in our study, thus we stated aims and objectives that we could strictly achieve with the kind of data we had: "We aimed to (1) describe the gut microbiota abundance and diversity patterns in wild populations of the three Mexican primate species; (2) evaluate the effects of phylogeny and geographic region as potential drivers of host microbial composition; and (3) assess the overall patterns of the gut microbiota in hybrid individuals and how distinct it is from the parental species."

Accordingly, based on the information we have and with which we could discuss the gut microbiota results obtained, we indeed described in the original version potential relationships with dietary preferences and overall locations of the studied species
(lines 434-449 in the clean (no track changes) revised version of the manuscript): “Life history variances and distinct feeding behaviors must also have an impact on gut microbial diversity; however, like in other studies with NHPs, correlation between diet and phylogeny complicates disentangling the factors directly affecting gut microbiota [22,33,38]. *Ateles* is mostly frugivore and *Alouatta* mainly folivore [41]. The higher proportion of Bacillota over Bacteroidetes present in *Alouatta* implies a better fermentation efficiency [84]. Indeed, howlers are adapted to a folivore diet which, as hind-gut fermenters, present large cecum and colon [85], slow food passage rates [41], and other physiological adaptations [86,87]. Accordingly, *Alouatta* microbial communities had more abundant and enriched taxa that have potential fermentative effects associated with folivory (i.e. Clostridiaceae) [22], while *Ateles* had a major diversity and abundance of Ruminococcaceae and Prevotellaceae, associated with degradation of sugars like those of fruits [17,21]. These patterns of gut microbial community composition were also present where *A. geoffroyi* and *A. palliata* individuals are sympatric (e.g. Playa and Mirador Pilapa localities at Los Tuxtlas region; Fig 1). Importantly, *Bifidobacterium* was previously described as a central agent in gut microbiota communities in *Alouatta* [51,88], but in the populations we studied it was only present in *A. geoffroyi*, albeit in low abundances. Altogether, these results show further evidence of both the significant effect of phylogeny on the microbial diversity and the close link between host physiology and gut microbiota in atelids.”

Similar aspects are further elaborated along other parts of the discussion.

We also discuss our findings regarding the hybrids and the microbiota patterns they present, and how they differ from the parental species, including the description of specific microbiota taxa and their potential relationship with the hybrids’ distribution (local environment), among others (lines 450-472). For this revised version we included important information regarding specific background knowledge for the hybrid system that we had not : “Interesting patterns were observed regarding the gut microbiota of hybrid individuals. They exhibit some unique taxa, a higher diversity of Bacillota compared to the parental species, and an intermediate overall diversity between the parental species, yet more similar to *A. pigra* (Fig 4). The latter is likely associated with the fact that only female hybrids are viable in these howler monkeys, resulting from the reproduction between *A. palliata* males and *A. pigra* females [54]. Hence, *A. pigra* mothers transfer their microbiome to their offspring and, therefore, the observed higher similarity of gut microbiota of hybrids to *A. pigra*.”

Additionally, we describe the potential connection of the species and the environment (up to what our information allowed for) at the population level, highlighting interesting microbiota patterns within species and comparing individuals from different localities, in the Discussion section entitled “**Environment, geography and gut microbial communities at the intraspecific level**”

We believe figure 3 is important; it allows the reader to easily visualize, in terms of number of ASVs (richness), how many taxa are shared among species/hybrids, as well as how many are unique to each one. We also describe in the text the most important associated information, namely what bacterial taxa are shared between the hybrids and their parental groups and also those that are unique to them (lines 450-472). This is complemented with Table S3, where the identification of each microbiota taxa per species is listed. Importantly, we not only provide data on bacterial taxa identity, but much more information about microbiota (abundance, different diversity indices, community composition, species turnover) that further highlight shared and restricted patterns among and within species and hybrids.

With all, we achieved the objectives of the study.

4. The study design was limited with regard to the resolution of taxonomic classification based on PCR amplification of the V4 region. The authors should include information regarding the number of reads that were discarded during processing due to chimeras etc.
Response: We appreciate the comment. In the revised version we include a table (Supplementary Table S2) where we describe the denoising statistics after quality filtering (number of reads that were discarded during processing due to chimeras, etc.) for each of the 40 samples.

5. The authors note the similarities and differences among the samples, but they do not offer much in terms of explanation for the observed differences. Also, there is little comparison to what was previously reported for these primates.
Response: We do not agree with the reviewer's appreciation. Within the limits of our study design, we do provide comparisons with what has been previously reported for the studied and for other primate species (see lines 405-417; 421-426; 445-449; 497-501 …). We also present our results in phylogenetic terms (phylosimbiosis) and discuss them in relation with these and other species (lines 418-426).

Importantly, to the best of our knowledge, this is the first study to describe gut microbial patterns in free-ranging spider monkeys; the few previous works have been done with captive animals, which we mention in the discussion as comparison (e.g. lines 485-487). Also, as Miller et al. (2021) emphasize, there is yet much to investigate regarding how hybridization affects the microbiome and how do hybrid organisms differ from their parentals. We provide the first gut microbiota information for hybrid atelids.

Regarding not offering much in terms of explanation for the observed differences, we provide a careful answer in numeral 3. above.

6. It does not appear that the authors have submitted their sequencing data to a public database to share with the research community. This should be done.
Response: We had not for the initial submission, but now they are publicly available in NCBI under the Bioproject ID PRJNA1189536.

We also comment here some points indicated in the email:
PLOS ONE Decision: Revision required [PONE-D-24-38914] - [EMID:2e3b39249bbe0878]

2. Thank you for stating the following financial disclosure:
If the funders had no role, please state: "The funders had no role in study design, data collection and analysis, decision to publish, or preparation of the manuscript."
Done

3. Please expand the acronym "CONAHCyT" (as indicated in your financial disclosure) so that it states the name of your funders in full.
Done

4. We note that you have referenced (unpublished data; Vázquez-Domínguez et al. in prep) on page 7, which has currently not yet been accepted for publication. Please remove this from your References and amend this to state in the body of your manuscript: (ie "Bewick et al. [Unpublished]")
Done

5. We note that [Figure 1] in your submission contain [map/satellite] images which may be copyrighted.
NO, the image/map is not copyrighted at all, as we indicated in the figure legend.
We briefly modified the legend to further clarified this:
Fig 1. Map ….. ….. "Information of geostatistical boundaries **was obtained from a public domain** [http://www.conabio.gob.mx/informacion/gis/] **and the map was created by us** using QGIS 3.22.10 software. "
